# Materials and Applications for Low-Cost Ceramic Membranes

**DOI:** 10.3390/membranes9090105

**Published:** 2019-08-21

**Authors:** Amanmyrat Abdullayev, Maged F. Bekheet, Dorian A.H. Hanaor, Aleksander Gurlo

**Affiliations:** Fachgebiet Keramische Werkstoffe/Chair of Advanced Ceramic Materials, Institute of Materials Science and Technology, Technische Universität Berlin, 10623 Berlin, Germany

**Keywords:** low-cost ceramic membrane, water filtration, inorganic membranes, oil-water separation, kaolin, fly ash, rice husk ash

## Abstract

In water treatment applications, the use of ceramic membranes is associated with numerous advantages relative to polymer-based filtration systems. High-temperature stability, fouling resistance, and low maintenance requirements contribute to lower lifecycle costs in such systems. However, the high production costs of most commercially available ceramic membranes, stemming from raw materials and processing, are uneconomical for such systems in most water treatment applications. For this reason, there is a growing demand for new ceramic membranes based on low-cost raw materials and processes. The use of unrefined mineral feedstocks, clays, cement, sands, and ash as the basis for the fabrication of ceramic membranes offers a promising pathway towards the obtainment of effective filtration systems that can be economically implemented in large volumes. The design of effective ceramic filtration membranes based on low-cost raw materials and energy-efficient processes requires a balance of pore structure, mass flow, and robustness, all of which are highly dependent on the composition of materials used, the inclusion of various pore-forming and binding additives, and the thermal treatments to which membranes are subjected. In this review, we present recent developments in materials and processes for the fabrication of low-cost membranes from unrefined raw materials, including clays, zeolites, apatite, waste products, including fly ash and rice husk ash, and cement. We examine multiple aspects of materials design and address the challenges relating to their further development.

## 1. Introduction

Industrialization, urbanization, and continued population growth have combined to bring about a sharp increase in the demand for water filtration capacity. In the last century, water consumption increased at more than twice the rate of human population growth, making water scarcity one of the most pressing challenges facing humanity [1]. The rapid industrialization in developing countries and the associated contamination of freshwater sources have further contributed to the need for increased water filtration and desalination capacity [2].

Partially pervious membranes are highly appropriate for filtration and desalination applications as their implementation requires significantly less energy relative to other separation methods, such as distillation and electrodialysis [3,4]. Most commercially available membranes are made from polymers. Polymeric membranes can be cheaply produced; however, as the result of fouling, such systems suffer from poor stability and high lifetime costs [5]. In contrast, the thermal and chemical stability exhibited by inorganic membranes based on ceramics or metals allows for the application of heat or chemical solvents for defouling processes, thus reducing operation costs [6,7]. However, high costs of raw materials typically used for ceramic membranes (alumina, zirconia, titania) and high energy consumption in sintering-based fabrication processes result in high production costs, and thus hinders large scale application of conventional ceramic membranes and limits their application to small scale systems [8,9]. Over the past few years, the use of low-cost raw materials as ceramic membrane precursors has been attracting increasing attention [10,11,12]. Studies have shown that worldwide, more than 2.5 billion people have limited access to clean water, predominantly in regions of lower economic development [13]. Low-cost ceramic membranes have the potential to provide a high volume filtration capacity that would facilitate the provision of clean and reliable water in poorer regions of the world.

To address large-scale water treatment challenges, there is a growing interest in the fabrication and application of low-cost ceramic membranes based on naturally occurring raw materials and waste products. The challenge in the fabrication of low-cost membranes relates to the obtainment of structures that exhibit the appropriate micro-scale pore structures to effectuate pollutant separation while maintaining sufficient mass transport and mechanical robustness. Numerous materials and processing approaches have been examined to achieve this aim using low-cost feedstocks, including unprocessed minerals, clays, and ash. The microstructures, durability, and filtration performance of membranes fabricated from impure raw materials sourced directly from mineral deposits or waste streams can be significantly altered through the use of pore formers, binders, fluxes, and other additives. The design of appropriate processing techniques, including thermal treatment, further governs the efficacy of the obtained membranes and influence overall system costs.

To date, the challenges facing the development of ceramic filtration membranes in low-cost processes have not been comprehensively reviewed. Some issues relating to the design and fabrication of diverse kaolin-based membranes were presented in [13], while the preparation and application of low-cost support membranes have been discussed elsewhere [14,15]. In this review, we present a comprehensive survey of water filtration systems based on cost-effective materials and critically examine their processing and implementation.

## 2. Materials

**Figure 1:**
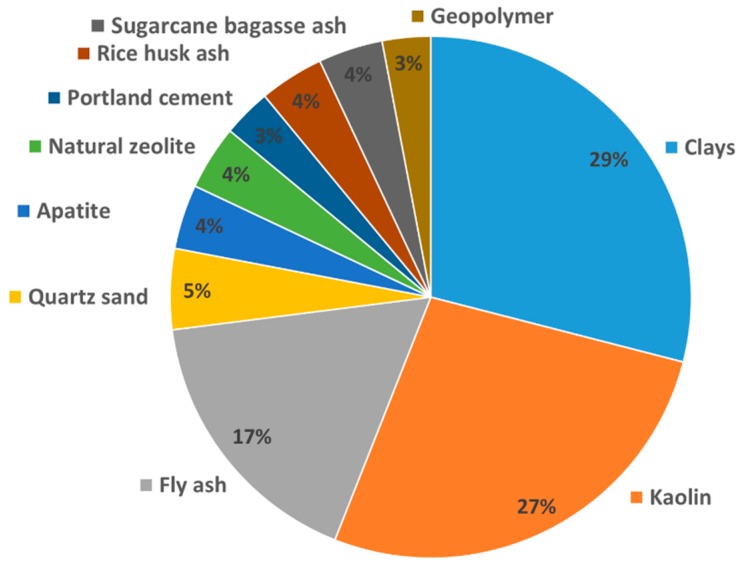
Representation of different raw materials in studies of low-cost inorganic filtration membranes.

A variety of low-cost alternatives to conventional materials like alumina or zirconia have been examined for use in water filtration. Those low-cost materials are either natural minerals (clays, zeolite, quartz, apatite) or waste from industrial production (ash). Based on the literature surveyed in this work, a breakdown of materials studied for the fabrication of low-cost membranes is shown in Figure 1. This figure demonstrates that clays are the most widely studied raw material for such applications, with fly-ash further playing a prominent role in this field.

Materials used as low-cost precursors in the fabrication of inorganic filtration membranes are predominantly based on unprocessed sources of alumina and silica. Obtaining high levels of performance while avoiding high processing costs remains a significant challenge in this field. Here we present a survey of the types of raw materials and processes that can be used in the design of water filtration systems and address the challenges that remain in implementing such materials in cost-effective ways.

### 2.1. Natural Minerals

With an emphasis on their implementation in water treatment membranes, here we review the use of widely available low-cost natural minerals composed of silica, alumina, silicates/aluminosilicates, and phosphates. These materials—such as clays, natural zeolites, apatites, quartz sand, and natural pozzolan—are obtained from natural sources and are implemented in membrane fabrication with no further processing steps besides crushing and grinding.

#### 2.1.1. Kaolin

There are numerous clay types on Earth distinguished by different chemical, mechanical, and physical characteristics. Predominant clay minerals include kaolinite, montmorillonite, and illite [16]. Naturally occurring clays have non-identical compositions that depend on localized formation conditions. Clays are widely available across the globe and require only minimal processing for membrane preparation. Hence, there have been significant efforts made towards the preparation of low-cost ceramic membranes using different types of clays [13]. Kaolin, the most widely found clay type, of which kaolinite is the main mineral form, is particularly suitable for membrane fabrication, owing to the pore structures and mechanical properties that can be achieved following thermal processing. Consequently, kaolin plays a central role in emerging low-cost membrane technology and merits a separate discussion.

Kaolin (Al_2_Si_2_O_5_(OH)_4_) is a type of widely occurring clay that has been studied as the basis of components in inorganic membranes, including support layers [17,18,19], microfiltration (MF) layers [20,21,22], and ultrafiltration (UF) layers [23]. Hubadillah et al. discussed kaolin membranes in detail in their recently published review paper, focused on the fabrication and application of kaolin-based low-cost membranes [13]. In particular, the lower thermal processing temperatures of kaolin, when compared with most conventional oxide ceramics and the morphology of decomposition products, namely spinel and mullite, are of key importance in the development of new membranes.

The thermal decomposition of kaolin and the formation of aluminosilicate phases is described in part by the reactions below [24]:

(1) $$Al_{2}O_{3}\cdot 2SiO_{2}\cdot 2H_{2}O\;\overset{400–700\;{}^{\circ}C}{\to }\;Al_{2}O_{3}\cdot 2SiO_{2}+2H_{2}O$$

(2) $$2\left(Al_{2}O_{3}\cdot 2SiO_{2}\right)\overset{925–1050\;{}^{\circ}C}{\to }2Al_{2}O_{3}\cdot 3SiO_{2}+SiO_{2}$$

(3) $$3\left(2Al_{2}O_{3}\cdot 3SiO_{2}\right)\overset{>1050\;{}^{\circ}C}{\to }2\left(3Al_{2}O_{3}\cdot 2SiO_{2}\right)+5SiO_{2}$$

Kaolin membranes have been studied both as composites and single-component systems. Kaolin membranes without any reactive additives presented only mullite (3Al_2_O_3_·2SiO_2_) and cristobalite (SiO_2_) phases following processing at temperatures higher than 1200 °C [21]. The use of yet higher temperatures further enhances mechanical strength through the formation of needle-like mullite structures, i.e., mullite whiskers, and densification of membranes [22,25]. However, it is possible to obtain different phase assemblages by including a various solid-state inorganic component with the propensity to react at high temperatures. For example, if an alumina source, like bauxite or pure alumina, is added, then cristobalite, formed during the metakaolin to spinel and spinel to mullite transformation steps, will react with alumina to yield an increased mullite content [26,27]. Various processes have used calcium and magnesium sources, such as naturally occurring calcite and limestone (CaCO_3_) [18,28], dolomite (CaMgCO_3_) [17], and commercial calcium carbonate [11], to obtain additional phases to mullite, such as anorthite (CaAl₂Si₂O₈) and cordierite ((Mg,Fe)_2_Al_4_Si_5_O_18_), which both influence membrane performance and form at relatively low temperatures compared to mullite.

Fabrication of membranes for water filtration applications using kaolin with or without additives is summarized in Table 1. Earlier research efforts employed significant levels of additives in fabrication processes [29,30,31,32]. Those additives not only take part in phase formation but through gas evolution, can further serve as pore formers. As a few examples, calcium carbonate assists pore formation through CO_2_ produced in decomposition, while quartz increases mechanical and thermal stability, and feldspar acts as a sintering aid, forming a fluxed glassy phase at low temperatures. Additionally, the inclusion of ball clay provides plasticity and strength to the green body in the early processing stages. It should be noted that a large number of constituents often involved in processing results in difficulties to identify the effects of each component on the final properties of the membrane. In more recent research, fewer additives are employed, and the use of kaolin raw materials solely with organic pore formers has emerged as a promising approach [33,34,35,36].

As a soft clay, the particle size of kaolin clay is sufficiently small for membrane fabrication. Nevertheless, further decreases in particle size can readily be achieved by milling. Most commonly, kaolin particles used in water purification membranes are between 15 µm [17] and 1 µm [18,35], with sintering temperatures ranging between 850 °C [23] and 1550 °C [27], with most processes using temperatures higher than 1150 °C [19,27,41], selected according to composition and desired pore size. Higher temperatures result in improved mechanical strength at the expense of reduced overall porosity.

**Figure 2:**
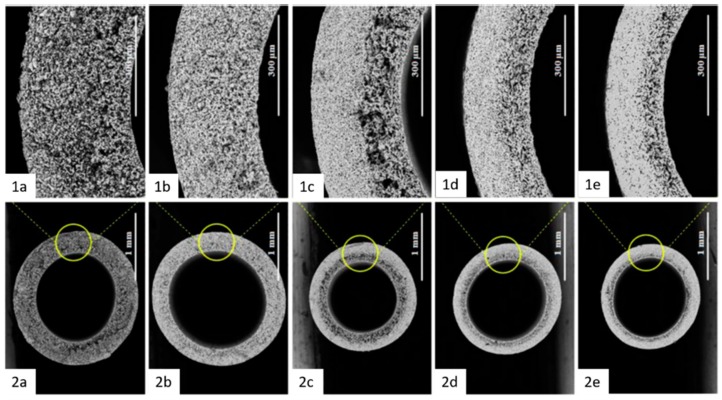
Hollow fiber membranes fabricated using kaolin at different sintering temperatures ((**a**–**e**) at room temperature, 1200 °C, 1300 °C, 1400 °C, and 1500 °C, respectively. 1a-1e enlarged cross section, 2a-2e normal cross section). At room temperature, hollow fibers have sponge-like pores; however, sintering at 1200 °C leads to densification by grain growth, and further increasing temperature leads to more densified membranes with smaller pores (adapted from ref. [22] with permission from Springer Nature).

The mechanical strength of kaolin-based membranes is governed by porosity and mineralogy, which are determined by additives and sintering temperature. Higher sintering temperatures lead to glass formation and improved bonding between ceramic particles, producing more stable membranes [22,29,35]. Usually, high temperatures, above 1200 °C, are required to obtain mullite-rich membranes with sufficient mechanical robustness for water treatment applications, as shown in Figure 2. However, the comparison of the mechanical strength of different membranes fabricated using kaolin, with or without additives, is rendered complex owing to the diversity of mechanical testing methods and sample geometries. For example, in many papers, three-point bending test is used [10,17,22,28,43], while in some cases, compression [21,33], biaxial flexural strength [26], and diametral compression tests are used [18,23,42]. It should be noted that, in general, while a three-point bending test is more appropriate for facilitating comparative analysis, mechanical stability tests should be designed according to the membrane shape (configuration).

To summarize, kaolin is cheaply available almost all over the world and can be applied in membrane technology as support or filtration layer. Fine powders can be produced from relatively soft kaolin, which is important for obtaining small pore sizes and high mechanical stability. Kaolin membranes without any reactive additives generally require sintering temperatures as high as 1300–1400 °C, which results in mullite formation, but alternative mineralogies can be obtained at a lower temperature in the presence of additives. Kaolin support layers have promising mechanical stability, and filtration layers have a pore size ranging from 0.1 µm to 1.2 µm, as well as porosity from 30% to 50%. All those make kaolin competitive material for low-cost membrane fabrication, especially hollow fiber membranes, as shown in Figure 1, with a high ratio of surface area to volume. Fabrication using kaolin reveals that kaolin-based membranes have comparable properties to commercial membranes.

#### 2.1.2. Other Clays

Further to kaolin, a broad variety of alternative clay types are also of interest towards low-cost filtration membranes. These clays include sepiolite, ball clay, bentonite, and attapulgite. Some properties of membranes fabricated using various clay types are presented in Table 2. The application of clays in water filtration has a long history [45]. However, earlier studies are related to water filtration by clay media in bed filtration systems rather than as filtration membranes and are therefore beyond the scope of this review.

The chemical composition of clay material according to the origin, particle property (clay composed of powder particles or fibrous clay, like attapulgite), and particle size of clays are diverse and do not follow any trend in membrane fabrication from clays, as can be seen from Table 2. It is worth mentioning that membranes with pore sizes as small as 3 nm [46], and flexural strength values up to 69 MPa are successfully achieved by cordierite membrane prepared from sepiolite clay [47].

**Figure 3:**
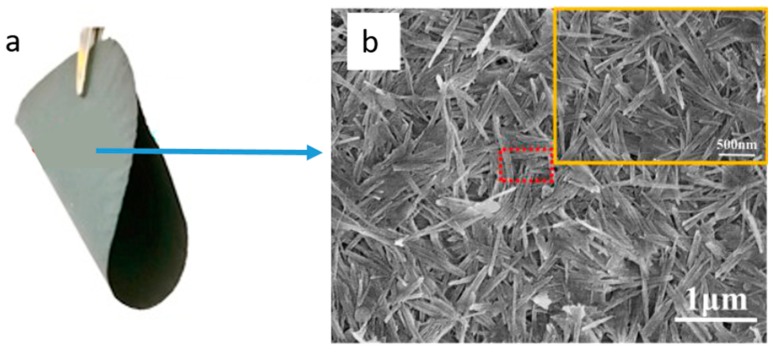
(**a**) Flexible attapulgite membranes; (**b**) SEM image of the membrane surface. Flexible membranes have been fabricated using fibrous attapulgite clay and sintering-free method (adapted from ref. [51] with permission from Elsevier).

Membrane preparation using attapulgite or palygorskite clay is a promising approach. Attapulgite is one of the most important naturally available fibrous clays with many attractive properties, such as a large specific surface area, excellent mechanical strength, high adsorptive capacity along with high chemical and thermal stability [50,72]. Moreover, membranes can be prepared from attapulgite without the need for high-temperature sintering [51]. Because of their fibrous composition, attapulgite-based membranes have competitive mechanical and filtration properties exhibiting pore sizes of around 12 nm and porosity above 60%, which makes them competitive with conventional membranes in UF applications [52]. Additionally, by adding long-chain polymers, such as polyvinyl alcohol (PVA), it is possible to obtain flexible fibrous membranes, as shown in Figure 3.

Clays are mixtures of minerals, and their composition varies with geographic origin, thus making comparative analysis challenging. Clay-based support layers prepared for use in filtration membranes generally exhibit a pore size between 0.3 µm [67] and 16 µm [68], porosity as high as 49% [69], and have shown flexural strength from 10 MPa [55] up to 69 MPa [47]. Using clays as raw material, it has been demonstrated that one can fabricate MF or UF active layers for suspended particle [61], oil droplet [65], dye [53], or heavy metal [20] removal applications. Results reported so far are supportive of the utility of clay in membrane fabrication in the context of sustainable water filtration technology.

#### 2.1.3. Zeolite Minerals

Naturally occurring zeolite minerals are mainly composed of hydrated aluminosilicates having a nominal composition of [(SiO_2_)(AlO_2_)_x_]M·yH_2_O. These natural materials exhibit a three-dimensional framework structure with nanoscale porosity [73]. Zeolites occur in many types of rocks but are most common in volcaniclastic sediments, and the largest and purest deposits are altered vitric tuffs [74]. They have a wide range of applications, such as in construction, water treatment, agriculture, catalysis, as well as medical applications [75]. There are many types of synthetic zeolites, which can be obtained by hydrothermal or other applicable methods [76]. However, here we focus only on naturally available zeolite materials, which can be sourced cheaply in large quantities and are thus of more direct relevance towards low-cost water filtration systems.

A significant portion of studies relating to the application of natural zeolites in environmental remediation applications examines the adsorption and ion exchange abilities of these minerals [77,78]. The application of this material in membrane systems offers an interesting pathway towards multi-functional water treatment materials, which combine filtration and adsorption mechanisms, in conjunction with facile defouling or regeneration processes.

Fabrication of membranes from natural zeolites involves steps of grinding, shaping, and sintering to obtain robust bulk materials of the desired aluminosilicate phases. An early study by Roque-Malherbe et al. used natural zeolite to fabricate porous support layers for membranes [79]. Subsequently, multi-layer ceramic microfiltration membranes were produced using different particle sizes of ground zeolites. Obtained membranes have shown pore sizes between 0.3 µm [80] and 1.1 µm [81]. By using starch as a pore-forming agent, pores as large as 6 µm can be formed [82]. The sintering temperatures of zeolites are relatively low, in the range 800–900 °C, following firing at 1000 °C unless pore-forming agents are used, and the naturally present pores in the zeolite structure may be filled with liquid phases, eliminating porosity [80]. The mechanical strength of zeolite-based membranes has not been comprehensively studied to date. Adam et al. reported that hollow fiber zeolite membranes presented up to 50 MPa strength by a three-point bending test [83]. Nevertheless, this is also good flexural strength compared to other hollow fiber membranes, e.g., kaolin-based hollow fiber membranes have flexural strength 15–63 MPa at a sintering temperature of 1200–1500 °C, where sintering temperature of the zeolite-based membrane is 1050 °C.

A study devoted to the fabrication of ceramic membranes using zeolite by Adam et al. presented hollow fiber ceramic membranes fabricated using an immersion precipitation method having separation-adsorption dual property, i.e., they can adsorb chromium and ammonia ions while providing filtration [83]. Zeolites are known for their superior ammonia ion absorptivity, which is advantageous for applications in the treatment of fertilizer contaminated water [84].

#### 2.1.4. Apatite

Apatites, having the nominal form Ca_5_(PO_4_)_3_(F,Cl,OH), are naturally occurring materials that have a wide range of applications in biomedical, chemical, pharmaceutical, environmental, and geological fields. As an example, apatite particles can be used to remove divalent heavy or radioactive metals from water by cation exchange process, where Ca^2+^ exchanges with target metal ions, such as lead ion [85]. There have been several studies related to the application of apatite in environmental technology to treat a variety of aqueous wastes and contaminated soils [86,87,88,89]. Apatites are not only able to efficiently adsorb metal contaminants but are also effective in the removal of anionic and cationic dyes by adsorption [90]. Unsurprisingly, the main focus of most studies has been the application of apatite in adsorption-based water treatment systems. However, a study by Masmoudi et al. [91] was among earlier works devoted to the application of apatite as a water treatment membrane. In this study, apatite was used as a low-cost raw material for the preparation of ceramic membranes. As with zeolites, apatite membranes are of interest towards the realization of dual adsorption-filtration functions, to facilitate the single-stage removal of multiple forms of water contaminants.

Apatites can be obtained in low-cost forms from naturally occurring deposits and can also be synthesized from waste materials [92]. Studies to date have shown that relative to synthetic materials, naturally sourced minerals are more cost-effective for the fabrication of apatite-based microfiltration membranes and support layers [91,93,94]. Results have revealed that membranes from natural apatite exhibit similar properties and performance to those exhibited by synthetic materials, with both having similar submicron pore sizes despite an initially varying particle size [94]. Nanofiltration membranes prepared from synthetic apatite have been reported with a pore size of 83 nm and porosity of 55%. These exhibited high permeability, 1011 l/h×m^2^ at a pressure of 0.8 bar, confirming the suitability of apatite-based membranes for water treatment processes [95].

Apatite-based membranes are predominantly applied on support layers of other materials, e.g., alumina support layer covered with apatite filtration layer; therefore, mechanical strength is seldom discussed. Only Masmoudi et al. [93] reported the preparation of flat support layers using apatite, with obtained materials exhibiting a flexural strength up to 30 MPa after sintering at a temperature of 1210 °C. This mechanical strength is comparable to other low-cost material-based membranes.

The wide range of temperatures used thus far for the sintering of apatite materials is noteworthy. Thermogravimetric analysis of phosphate studies indicates a first weight loss between 25 °C and 250 °C corresponding to the desorption of water. A second mass loss between 250 °C and 450 °C corresponds to the elimination of organic matter. The weight loss between 450 °C and 1100 °C can be attributed to the decomposition of mineral carbonates present in the natural apatite. However, apatite membrane preparation studies have used a broad range of different temperatures, such as 600 °C [91], 750 °C [96], 900 °C [95], and 1150–1200 °C [93]. This diversity in processing methods demonstrates the need for further studies to establish optimal processing conditions towards functional apatite-based membranes.

#### 2.1.5. Quartz Sand

Natural quartz sand is a sedimentary rock that consists of crystalline silicon dioxide in the form of quartz (SiO_2_). It is highly resistant to both mechanical and chemical weathering. Hence, quartz is among the most abundant and widely distributed minerals found at Earth’s surface [97]. Geological processes have occasionally deposited sands that are composed of almost 100% quartz grains. These deposits have been identified and produced as sources of high purity silica sand [98]. These sands are of particular value in the glassmaking industry.

Sand has a long history in water purification applications, having been used since 1829 for the production of slow sand filters (SSF), an earlier industrial water treatment process [99]. As the name refers, this water cleaning method is slow and has many drawbacks, that is why nowadays slow sand filters mainly exist in developing countries [100].

Silica sands are noted by their chemical homogeneity and high purity in naturally occurring forms. Several studies have been conducted dealing with the preparation of water filtration membranes from natural quartz sand [40,101,102,103,104,105]. In the fabrication of quartz sand-based membranes, a binder phase is essential to facilitate the cohesion of quartz particles to one another and thus ensure adequate performance and robustness. In certain cases, the selection of binders can result in the obtainment of functional quartz sand-based membranes using processing temperatures as low as 600 °C [103]. However, in general, the additive assisted sintering of quartz requires temperatures of at least 800 °C to get support layers with desired mechanical strength [101,104]. In the absence of sintering additives, temperatures higher than 1040 °C are required [102].

**Figure 4:**
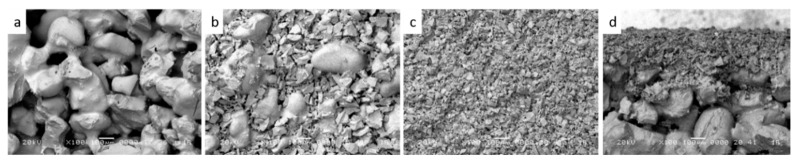
SEM image of quartz sand-based membrane composed of three layers. Surface image of (**a**) Support, (**b**) intermediate, and (**c**) active microfiltration layer; (**d**) cross-section of the whole membrane. Using different fractions of natural quartz sand, it is possible to fabricate support and the active layer of membranes (adapted from ref. [101] with permission from Elsevier).

When we compare the mechanical properties of supports obtained from mixtures mainly composed of quartz sand, it is worth to mention that they have acceptable strength at relatively lower temperatures, generally, between 12–20 MPa with sintering temperature around 1200–1300 °C. Studies have shown that using quartz sand of different particles sizes, as shown in Figure 4, it is possible to fabricate microfiltration membranes with pore size 10 µm and even ultrafiltration membranes using fine powders, with a pore size as low as 10 nm.

Based on a survey of related literature, it can be concluded that on the basis of naturally and cheaply available quartz sands, it is feasible to prepare support, microfiltration, and ultrafiltration layers for water treatment membranes. However, further detailed research is necessary to fully understand the effect of sintering temperature, particle size, additives on pore structure, and mechanical properties of membranes.

#### 2.1.6. Natural Pozzolan

The term pozzolan is used generically to define materials, which have constituents that at ambient temperature combine with lime in the presence of water to form permanently insoluble and stable compounds that behave like cement, and that is why natural occurring pozzolan minerals are mainly used in cement industry [106]. Rather than forming a cement, Achiou et al. sintered pozzolan material at 950 °C, to obtain microfiltration membranes with porosity around 30% and pore size 2–3 µm, which were successfully applied for treating wastewater from textile industries [107]. Later, the effect of starch as a pore-forming agent was studied and revealed that it is possible to increase porosity up to 50% in these low-cost membranes [107]. In other studies by Achiou et al., tubular pozzolan multi-layer ceramic membranes were prepared for pretreatment of seawater in desalination processes [108], as a support layer for graphene oxide composite membranes [109] and as a support layer for synthetic zeolite membranes [110]. Results thus far have been promising, motivating the further investigation of pozzolan-based membranes. Nevertheless, in all published work on this topic, a sintering temperature of 950 °C has been used. Phase change behavior with temperature should be investigated to see if more durable and valuable phases occur at higher temperatures or how porosity and pore size distribution change with thermal treatment and densification.

#### 2.1.7. Bauxite

Bauxite is a sedimentary rock consisting of chiefly aluminum minerals, and its worldwide estimated reserve is 55–75 billion tons [111]. At high temperatures, the main residue of bauxite is alumina with some other iron and silicon oxides. Alumina widely used in conventional membrane preparation and bauxite, as an alumina precursor with some impurities, is also a potential candidate for membrane industry.

As an alumina source, bauxite is added to kaolin-based and fly-based membranes, and this type of membranes are discussed in Section 2.1.1 and Section 2.2.1. Here, we have discussed papers which used bauxite alone to prepare membrane. Unfortunately, there are only two papers published up to date related to bauxite membrane [111,112]. Both papers reported the preparation of hollow fiber membrane using phase inversion by immersion method and then sintering at high temperature. It is surprising that membranes obtained by Li et al. presented flexural strength between 24–183 MPa at sintering temperatures from 1200 °C to 1350 °C, which is quite better than compared to pure alumina hollow fiber membranes [112]. However, Esham et al. reported flexural strength of membrane to be between 5–70 MPa despite higher sintering temperature, i.e., from 1250 °C to 1450 °C [111]. The main reason for this difference could be the chemical composition of starting bauxite, wherein former work iron oxide content was around 2.7 wt. % but in the latter, it was 22.8 wt. %. Effect of sintering temperatures on the mineralogical composition was not presented, but authors stated that when bauxite was sintered up to 1600 °C, which was not used in membrane fabrication, the main phases were mullite and corundum.

Porosity and pore size of obtained membranes are not studied very well; however, according to SEM images, morphological properties are also competitive to pure alumina counterpart.

Generally, the idea of preparing membranes from sole bauxite is promising and effective because it eliminates extra procedures, such as first obtaining alumina and then preparing a membrane. However, extensive investigations are required to discuss the effect of sintering temperature, fabrication techniques, and particle size of starting bauxite on mineralogical composition, morphology, and water filtering abilities of membranes containing only bauxite.

### 2.2. Waste Materials (Ashes)

Utilization of waste materials and by-products from different industries is a key focal point in research towards sustainable materials development. Coal-fired power plants and agricultural industries often produce large volumes of ash as by-products, which pose a significant environmental problem if they are not handled appropriately [111]. However, these so-called waste materials, which include significant silica content, offer various pathways for valorization. Fly ash, rice husk ash, and sugarcane bagasse ash are produced in high volumes worldwide, and they can be used as raw materials for technological applications, including membrane technology [112].

#### 2.2.1. Fly Ash

**Figure 5:**
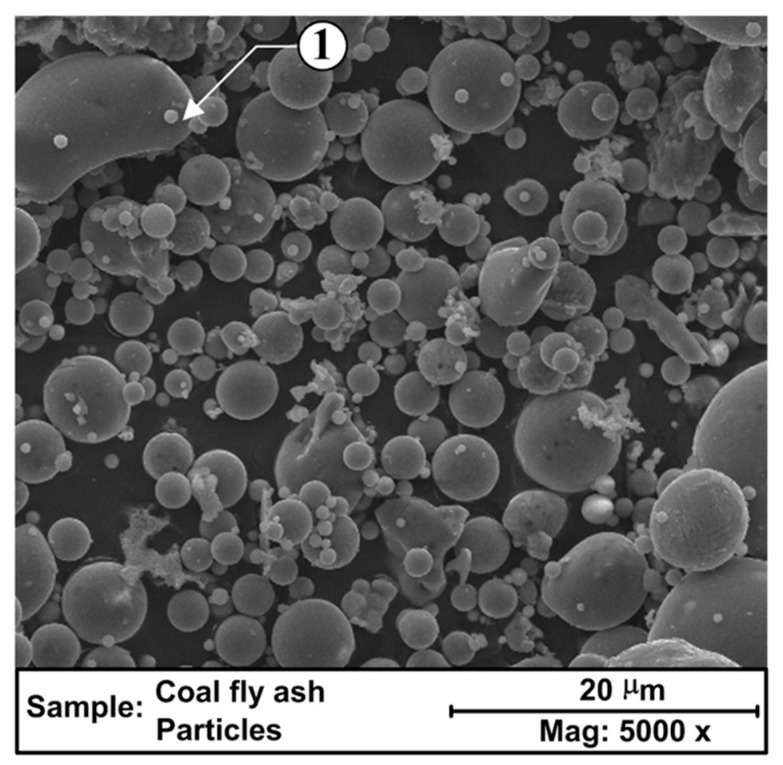
SEM image of typical fly ash composed of mainly spherical particles and 1- some irregularly shaped grains (reproduced from ref. [113] with permission from Elsevier).

Being a by-product of coal combustion, fly ash is among the most abundant waste materials produced and, despite being implemented in various industries, including concrete and paving, its safe disposal remains problematic [111]. The use of fly ash as a raw material in membrane fabrication offers a pathway towards low-cost water treatment solutions. The spherical particle geometries, as shown in Figure 5, are lying between 1 and 100 microns in diameter, and the silica-rich composition of readily available fly-ash is conducive to the fabrication of porous materials containing phases of cordierite, mullite, and anorthite, according to the additives used.

Fly ash is a heterogeneous material, with major components of SiO_2_, Al_2_O_3_, Fe_2_O_3_, and CaO [114]. The exact composition of fly ashes varies depending on the origin of the coal from which it is derived. Fly ash is composed predominantly of a glassy phase alongside phases of quartz, mullite, maghemite (γ-Fe_2_O_3_), hematite (α-Fe_2_O_3_), periclase (MgO), rutile (TiO_2_), gehlenite (Ca_2_Al_2_SiO_7_), and anhydrite (CaSO_4_) [115]. At higher temperatures, glassy materials can crystallize to form further crystalline products, such as esseneite (CaFeAlSiO_6_), mullite (3Al₂O₃2SiO₂), and cristobalite (SiO_2_).

The mineralogical composition of membranes prepared from fly ash exhibits tremendous variation, depending not only on the composition of the raw material but also on the sintering temperatures and additives used.

In the absence of additives, the phases formed in fly ash derived membranes are dependent only on sintering temperature. Following sintering at 800 °C, materials are composed of quartz, mullite, anhydrite, gehlenhite, and hematite [116]. However, at relatively higher temperatures, e.g., above 1200 °C, mullite becomes the main phase alongside other phases, such as anorthite, hematite, cristobalite [117].

By combining fly ash with bauxite, membranes exhibiting improved mechanical and chemical stability can be obtained, composed of mainly mullite, which has in turn very interesting properties like low expansion coefficient, good high-temperature strength, creep resistance, low density, and good chemical inertness [118,119,120,121,122,123]. The simple sintering of fly ash with bauxite presents a cost-effective route to synthesize mullite porous ceramics. Addition of bauxite, an alumina source, to fly ash is based on the composition of 3:2 mullite. Bauxite containing fly ash requires higher temperatures for sintering, around 1200–1500 °C, while fly ash without any additives requires 800–1000 °C, while additives like kaolin require 1200–1350 °C, and dolomite requires 1100–1200 °C.

**Figure 6:**
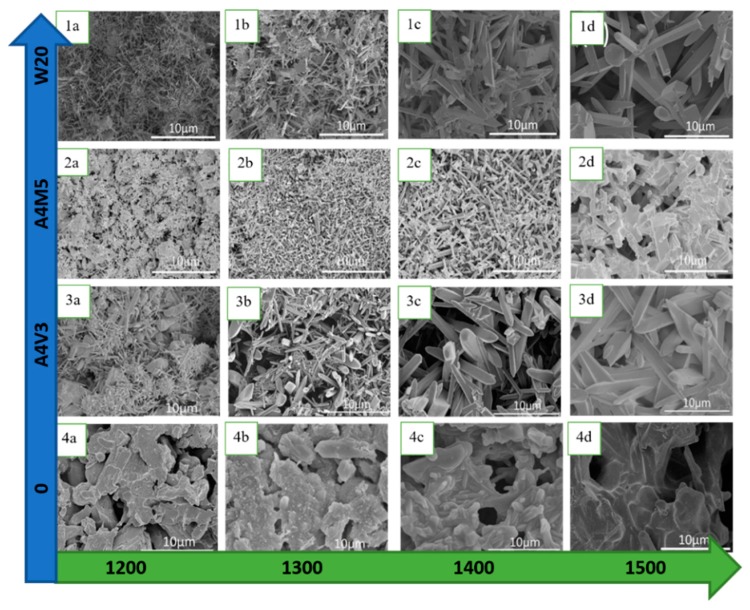
Formation of mullite from fly ash and bauxite at different temperatures with/without various sintering aids: 1a, 1b, 1c, 1d contain WO_3_, 2a, 2b, 2c, 2d contain MoO_3_, 3a, 3b, 3c, 3d contain V_2_O_5_, and 4a, 4b, 4c, 4d without sintering aid. Increasing sintering temperature leads to higher densification. Without any additives, platelet-like mullite crystals are formed; however, sintering aids promote the formation of mullite whiskers at lower temperatures, especially V_2_O_5_ (adapted from ref. [120,121,123] with permission from American Chemical Society and Elsevier).

When pure fly ash is sintered, at temperatures around 1200 °C, one of the main components formed is cristobalite. However, if bauxite is combined with fly ash, then that cristobalite (SiO_2_) will react with corundum (Al_2_O_3_) to form mullite, which is called secondary mullitization process [118]. The sintering temperature can be lowered by the use of sintering aids to lower the secondary mullitization temperature; however, the use of these additives can have adverse effects on pore size and porosity of membranes because of lower high-temperature viscosity [119]. Further studies toward the influence of other sintering aids, such as AlF_3_ and V_2_O_5_ [120], MoO_3_ [123,124], WO_3_ [121], demonstrated the utility of additives in directing the formation of needle-like mullite whiskers, which has increased open porosity as well as high water permeability. The effect of mentioned sintering aids on the mullite formation is presented in SEM images in Figure 6, where results are significantly different from mullite formed without any aid.

In various studies, dolomite [125], kaolin [126], carbonates [127,128], and quartz [129,130] have been added to fly ash and sintered to obtain different mineral phases with interesting properties, such as cordierite, anorthite, gehlenhite. When CaO exists in a system, from calcium carbonate or dolomite, according to the CaO-SiO_2_-Al_2_O_3_ ternary phase diagram, the formation can take place at temperatures around 1250 °C. In the same way, if fly ash and dolomite are mixed, MgO from dolomite also react with fly ash components and forms cordierite mineral at temperatures above 1100 °C [125,131]. The sintering temperature of membranes containing the aforementioned additives is lower than bauxite containing membranes.

As can be seen from Table 3, membranes prepared from fly ash have pore sizes that render them suitable for use as microfiltration (MF) and support layers. However, the majority of studies have focused on the preparation of support layers from fly ash because of the high mechanical strength of post-sintering minerals. Generally, membranes fabricated from fly ash have a pore size from 0.18 µm to 7.28 µm, see Table 3. When fly ash is combined with zeolites, it is possible to prepare nanofiltration membranes, as presented by Zhu et al. [132], where pore sizes are nanoscale. Dong et al. [118] and Zhu et al. [123] both used very similar raw materials, preparation techniques, and sintering temperatures. It is surprising, therefore, that the pore size of obtained membranes differed almost tenfold, i.e., 0.93–2.2 µm in former and 0.18–0.26 µm in latter. The only difference in these works is the use of a sintering aid, which facilitated the formation of mullite at lower temperatures.

It should be noted that to obtain fly ash-based membranes with pores smaller than 1 µm, particles smaller than 5 µm are necessary. Otherwise, pores may be too large (2–7 µm) [133], or special sintering aids may be required to obtain smaller pores [121]. The overall porosity of a membrane is also as important as its pore size, as this parameter directly affects water permeability and the mechanical durability of membranes. Generally speaking, support layers should have a porosity higher than 45–50%, and fly ash support, as well as microfiltration membranes, have a porosity between 30% [122] and 52% [121]. Pure fly ash membranes have a flexural strength of up to 22 MPa while adding bauxite increases sharply to 65 MPa [118]. It is also possible to get a very durable hollow fiber membrane from fly ash [122].

Overall, recent studies have demonstrated the merit of utilizing waste fly ash in membrane fabrication. Whether fly ash derived materials are used as support layers or microfiltration layers, in this material, the mullite dominated microstructures offer valuable pathways towards high performance, low-cost systems. In particular, bauxite-fly ash combinations have shown tremendous promise towards the design of multi-phase filtration layers with well-controlled porosity without incurring significant processing or raw-materials costs.

#### 2.2.2. Rice Husk Ash

Rice is the world’s second most important cereal crop following only corn. Nearly 482 million metric tons of husked rice was produced in 2018 worldwide [135]. Rice husk (RH) is a coating of rice grains. It is formed from hard materials, 70–80% organics, and 20–30% inorganics, including silica, and RH is mostly indigestible to humans. Therefore, the husk of rice is removed in processing steps, accounting for 20 wt. % of raw grain weight. Due to its high calorific value, RH can be used as fuel in boilers for energy production [136]. The burning of rice husk generates rice husk ash (RHA) as waste, accounting for roughly 25% of the rice husk weight. As a result, the production of 100 kg of rice incurs the production of 25 kg of rice husk, which when burned will yield 6.25 kg of RHA, of which around 5 kg is amorphous silica. In other words, the annual worldwide production of 482 million metric tons has the potential to yield 20 million metric tons of amorphous silica as raw material for membrane technologies and other applications.

There are different methods for the extraction of silica from RH. The properties of silica obtained from rice husks depend on both the feedstock and extraction process [136]. RHA has been intensively investigated as an adsorbent for the removal of heavy metals from wastewaters [137].

**Figure 7:**
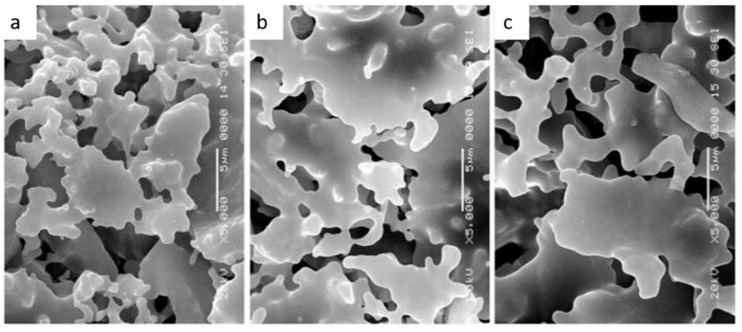
SEM image of the surface of RHA (rice husk ash) derived silica-based hollow fiber ceramic membranes at different sintering temperatures: (**a**) 1200 °C; (**b**) 1300 °C; (**c**) 1400 °C. Increasing sintering temperature leads to higher shrinkage and lower porosity (adapted from ref. [140] with permission from Elsevier).

In an earlier study, Bhavornthanayod et al. used RHA as a silica source for the preparation of zeolite membranes on porous substrates by a sol-gel method [138]. But, no porosity or permeability data was presented. Serra et al. added alumina to RHA to fabricate 3:2 mullite ceramics and presented that these porous or dense mullite ceramics exhibit attractive performance towards membrane applications. Hubadillah et al. used RHA to prepare hollow fiber membranes with dual adsorption-separation functions. RHA-based hollow fiber membranes facilitate the effective removal of heavy metals with high separation efficiency up to 99%, as well as very good average pore size and porosity of 1.2 μm and 36.7%, respectively [139]. Sintering temperatures are moderate and typically lie between 1200 °C and 1400 °C. Increasing sintering temperature leads to increased strength and grain size, but lower porosity, as shown in Figure 7.

Later Hubadillah et al. extracted silica from RH and fabricated silica-based hollow fiber membranes with a 3-point bending strength of 71.21 MPa, pore size with a broad peak of 0.55–2.3 μm, and porosity of 43.1% [140]. In their follow-up study, the hydrophobic surfaces were imparted on hollow fiber membranes for water desalination. Results are promising with excellent water flux (38.2 kg/m^2*^h) and salt rejection (>99.9%).

#### 2.2.3. Sugarcane Bagasse Ash

In the sugar and alcohol industries, sugarcane is crushed to extract the juice, and residual fibrous material is called bagasse. Sugarcane bagasse has various potential applications like producing textiles, paper, and pressed wood [141]. However, in developing countries, it is mostly used as an energy source for boilers. Sugarcane bagasse ash (SCBA) is a residue resulting from the burning of bagasse [142]. Waste SCBA can be obtained in enormous quantities as a by-product from combustions in boilers, from 1000 kg of sugarcane 250 kg bagasse fiber, and burning this bagasse fiber produces 6 kg of ash [142]. Compared to RHA, SCBA contains up to 20% of alumina beside silica [143].

Directly applying SCBA as raw material for membrane fabrication has been presented in works of Jamaluddin et al., where they used ash to prepare hollow fiber membranes with phase inversion and sintering techniques [144]. Obtained hollow fiber membranes have comparative properties with other hollow fiber membranes from fly ash, rice husk ash in literature. Authors then changed the surface hydrophilicity of membranes, consequently, got superhydrophobic and superoleophilic membranes for oil-in-water emulsion treatment [145,146]. Results are promising with an oil/water separation efficiency of 99.9%.

### 2.3. Cement

Most of the research efforts towards low-cost ceramic membranes have used naturally available minerals in conjunction with high-temperature processing steps [29,30,55,147], which generally incurs significant energy costs. To further decrease the costs of ceramic membranes, there is interest in developing sintering-free preparation routes.

Sintering aims to give mechanical strength to membrane green bodies through fusion and densification. Ordinary Portland cement and geopolymer cement achieve mechanical strength without sintering. In this section, membranes fabricated using Portland cement are discussed first, followed by membranes prepared by using geopolymers, a newer kind of cement.

#### 2.3.1. Portland Cement

Portland cement is used for construction purposes and is characterized by good strength as well and well-developed production technology [148]. Cement contains numerous chemical constituents, the most important of which are tricalcium silicate (3CaO·SiO_2,_ denoted as C3S), dicalcium silicate (2CaO·SiO_2_, C2S), tricalcium aluminate (3CaO·Al_2_O_3_), and alumino-ferrite phases (4CaO·Al_2_O_3_·Fe_2_O_3_) [149]. When water is mixed with cement, C3S rapidly reacts to produce calcium silicate hydrate gels (C-S-H) and calcium hydroxide as the resulting pH quickly increases to over 12. Another important reaction is the reaction of C2S with water, which will also produce C-S-H gels and calcium hydroxide; however, C2S is much less reactive and reacts slowly compared to C3S. These hydration reactions are given in the Equations (4) and (5). The other reactions upon addition of water or later by the formation of new phases occur with aluminate phases, but these reactions do not contribute to the strength of cement, that is why they are neglected here [150].

(4) $$2{\mathrm{Ca}}_{3}{\mathrm{SiO}}_{5}\;+\;7H_{2}O\;\to \;3\mathrm{CaO}\cdot 2{\mathrm{SiO}}_{2}\cdot 4H_{2}O\;+\;3\mathrm{Ca}(\mathrm{OH}{)}_{2}\;+\;173.6\;\mathrm{kJ}$$

(5) $$2{\mathrm{Ca}}_{3}{\mathrm{SiO}}_{4}\;+\;5H_{2}O\;\;\to \;3\mathrm{CaO}\cdot 2{\mathrm{SiO}}_{2}\cdot 4H_{2}O\;+\;\mathrm{Ca}(\mathrm{OH}{)}_{2}\;+\;58.6\;\mathrm{kJ}$$

The main focus of researchers was using Portland cement as a constituent in mortar, concrete. For example, there are thousands of works to increase the mechanical strength, workability, and chemical stability against leaching chemicals of concrete, mortar [150,151]. To the best of our knowledge, there was not any research devoted to the application of Portland cement as membrane until 2014. Wang et al., for the first time, used ordinary cement to prepare membranes for ozone disinfection of water [152].

While cement offers a cheap and abundantly available raw material, obtaining membranes with appropriate porosity levels based on cement is a key challenge. The pore size and porosity of cement-based structures mostly depend on the water to cement ratio. When cement hydrates, it produces two types of pores, nanoscale pores, known as gel pores, which exist between C-S-H sheets having equivalent diameters smaller than 10 nm [153], and capillary pores, which exist in spaces between cement particles that are not filled by hydration products, which have diameters of up to 1 µm [154]. Gel pores are very small and not connected, so they are not active in the filtration processes. Capillary pores are also small compared to conventional support layers; moreover, to obtain more capillary pores, i.e., increase open porosity of cement, we need to add excess water which in turn decreases mechanical stability [155].

Conventional methods to generate pores in the membrane fabrication process, such as the inclusion of dispersed organic additives followed by pyrolysis, are not relevant for cement materials [156]. For this reason, increasing porosity in cement-based membranes necessitates pore formation methods without high-temperature calcination.

**Figure 8:**
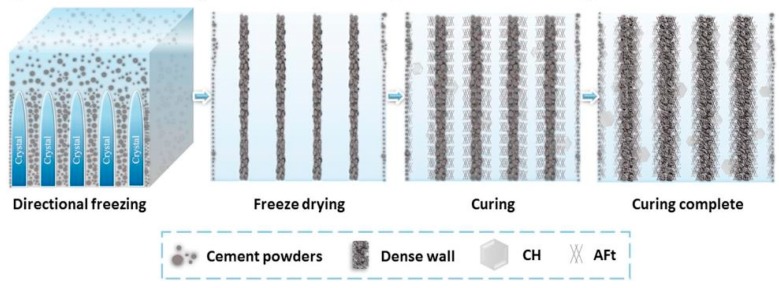
Fabrication of cement membrane using the freeze-casting method (reproduced from ref. [159] with permission from Elsevier).

Freeze-casting or ice-templating can also be applied to fabricate porous cement membranes with ordered pores, as shown in Figure 8 [157]. A freeze-casting process is a promising and environmentally friendly method for preparation of porous materials, as it can produce interconnected pores, e.g., aligned pore channels on a scale of several microns to hundreds of microns, which will offer interesting properties [157]. The different solvent used to prepare the slurry for freeze-casting will produce different pore structures [158]. Cement membranes fabricated, using water and tert-butanol as a solvent, have presented different pore size, porosity, and mechanical strength [159].

In their latest paper, Dong et al. [160] presented Portland cement membranes fabricated through the ice-templating technique with competitive porosity, 50–60%, and pore size ranging from 0.02 µm to 45 µm. However, the compressive strength of samples was around 15 MPa after 28 days of hydration of cement, which is quite low compared to normal hardened cement paste [161]. Of course, porosity is the thing that directly affects the strength of the membrane. Additionally, it is worth to mention that, authors mixed water and cement, then incubated it for 12 h to prepare a homogeneous slurry. However, the hydration reaction of Portland cement with water starts immediately after mixing, and that is why ASTM C-94 requiring the discharge of the concrete shall be completed within 90 min after the introduction of the mixing water [162]. Authors just prevented settling of cement while ball-milling 12 h, but hydration continued and, consequently, mechanical strength decreased.

The low-cost of raw materials, rapid and simple fabrication methods, low maintenance requirements, and potentially competitive performance are all factors that contribute to the attractiveness of cement-based water filtration membranes. Further work is required in this field to improve mechanical strength and establish appropriate processing methods for these materials.

#### 2.3.2. Geopolymer Cement

Geopolymers are a new class of material that have competitive properties compared to traditional Portland cement. Similar to ordinary Portland cement, geopolymer cement is also a binding system that hardens at ambient temperature [163]. With the reaction of alumina silicate material with soluble sodium or potassium silicates, water produces long-range, covalently bonded, amorphous polymers or networks, which is called geopolymer [164]. However, amorphous networks rearrange at a higher temperature, such as 600 °C or 1200 °C, to form crystalline phases, which have higher strength [165].

As with Portland cement membranes, geopolymer membranes are only seldom studied [166,167,168]. Geopolymer membranes offer advantages of simple low-cost fabrication in a sintering-free technique using low-cost materials. Membranes obtained from metakaolin (calcined kaolin clay) and sodium silicate present competitive properties, such as pore size between 20 nm and 100 nm, as well as 100% rejection of nanoparticles [166]. The mechanical strength of geopolymer membranes depends on the molar ratio of H_2_O/Na_2_O and SiO_2_/Na_2_O. Xu et al. reported the mechanical strength of geopolymer membranes measured by a compressive test to be between 10–50 MPa [166]. This is a good result compared to other low-cost membranes.

**Figure 9:**
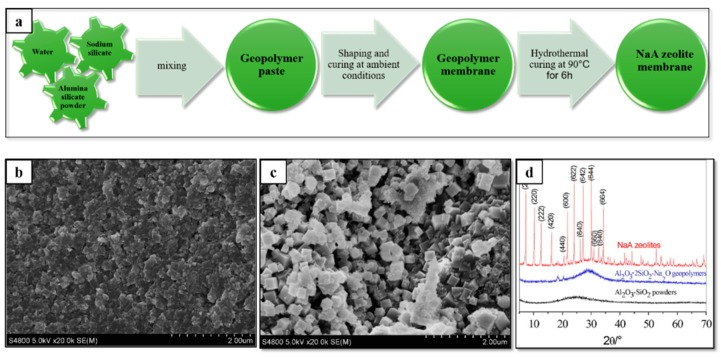
Geopolymer membranes from cheaply available and non-hazardous materials. Hydrothermal curing of geopolymer produces self-supporting zeolite membranes. (**a**) fabrication route; (**b**) cross-section of geopolymer membrane; (**c**) cross-section of zeolite membrane obtained from geopolymer; (**d**) XRD patterns confirming the formation of zeolite membranes (adapted from ref. [167] with permission from Elsevier).

Furthermore, geopolymer membranes can be transformed into a zeolite membrane by hydrothermal process, see Figure 9. This zeolite membrane has very interesting properties for organic solvent dehydration or desalination of salty waters [167]. Additionally, geopolymer inorganic membranes are efficient in the removal of a Ni^2+^ ion from wastewater. The combined actions of adsorption and rejection make geopolymer membranes potential industrial wastewater filtration membranes of future [168].

Geopolymer can be obtained from a wide range of precursors, such as calcined clays, volcanic rock, blast furnace slag, and fly ash. The use of geopolymer materials offers a route towards low-emissions membrane technology with potential applications in low-cost water treatment [169].

## 3. Application Areas of Low-Cost Inorganic Membranes

Membrane technology has a wide application area because of its simplicity and efficiency of the cost compared to other traditional filtration/separation process as distillation, absorption, or adsorption [4]. Rising human population, water pollution as the result of industrial growth, and limited freshwater resources, all these desire new technologies to treat water before discharge and/or before using them [1,170]. Membrane technology is, without a doubt, one of the best methods to treat wastewater and, of course, desalination of seawater.

**Figure 10:**
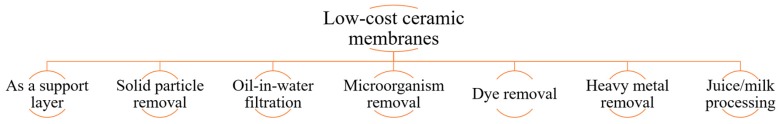
Various application areas of low-cost ceramic membranes.

Low-cost membranes prepared from various naturally available or waste materials have also wide application in the water treatment process [15,171]. According to the publications so far, applications of low-cost membranes can be classified as follow: as a support layer for further membrane preparation [66,68,132,172], microfiltration of suspended solid particles [36], oil droplets [32], dye from textile industry [116], bacteria [11,49], humic acid [126], ultrafiltration of uranium and other heavy metals [20,35,66], see Figure 10.

Most studies relating to the fabrication of low-cost membranes involve the fabrication of a support layer, which is then covered with an active layer for further MF, UF, or even desalination applications [173]. One of the most frequently studied water treatment problems for which low-cost membranes are applied is the separation of water-oil mixtures.

The hydrocarbon concentration in oily wastewater from various industries usually ranges between 50–1000 mg/L, which is considered hazardous and demands treatment with reduction of oil concentration to the tolerable limit, 10–15 mg/L, before discharge to the environment [174].

A lot of methods to treat oily wastewater are already adopted, such as chemical de-emulsification, coagulation, air flotation, and gravity settling; however, those methods have their drawbacks, i.e., while they solve one environmental problem, they produce another issue by secondary generation of pollutants; cost and energy efficiency are disadvantages as well [175]. Moreover, the mentioned methods are not efficient when oil concentration is low [176]. On another hand, membrane separation technology is gaining popularity in the last years because of its separation ability and cost efficiency [177]. Thermal and chemical stability, higher selectivity, and other important properties of ceramic membranes made them preferable against their polymeric counterparts [49,178]. Compared to conventional ceramic membranes, low-cost membranes also have a competitive oil separation ability, see Table 4.

For a given separation membrane, oil-water separation efficacy depends on the droplet size and concentration of the oil. For a constant pore size, ~1.30 µm, when the droplet size is changed from 0.92 µm to 6.9 µm, oil removal rates change from 85% to 99.2% [11,129,179]. The mean pore size of a membrane has a great effect on oil separation. Most commonly, a smaller pore size results in higher oil filtration efficiency. However, this trend is not universal, for example, it has been found that when the pore size is increased from 0.5 µm to 1.32 µm, the oil separation also increases from 96% to 99.2%, where droplet size and concentration are the same [129,179]. These could be explained by the hydrophilicity of the membrane surface. The high oil rejection of membrane despite its large pores is the result of surface properties. Increasing the hydrophilicity, using surface grafting or hydrophilic material, can improve oil rejection of membranes with relatively large pores, which in turn leads to higher water permeability and fastens the filtration process [25].

A key aspect of low-cost ceramic membranes is the safety concerns relating to the secondary contamination of water while filtering. For example, according to the origin of coal, fly ash can contain different amount of radioactive elements (such as U, Th, Ra, Rn) [184] and heavy metals (such as Pb, Ni, Cr, Mn) [185]. Not only fly ash but also other materials discussed above (clays [186,187], cement [188]) may also contain mentioned contaminants according to their sources. Those heavy metals and/or radioactive elements can leach out from membranes and can contaminate water again with more dangerous pollutants. Unfortunately, leachability or dissolution of toxic elements from membranes is not studied almost at all. However, Wang et al. [152], Zhu et al. [122], and Dong et al. [189] tested effluent of cement-based, coal fly ash, and cordierite-based membranes, respectively. They found that heavy metals are far below the allowed limits of standards. But, as fly ash composition depends on its origin, composition and leached products of fly ash-based membranes from another origin can have different results as well. That is why it is necessary to investigate the membrane effluent to ensure the safety of the low-cost membranes.

It is presented in Table 4 that membranes prepared, using kaolin, fly ash, clay mixtures, sugarcane bagasse ash, and attapulgite clay, have comparative oil separation ability as membranes prepared using conventional materials, alumina, zirconia, titania. All these studies reveal that wastewater treatment from various industries could be achieved in a cost-efficient way by using natural raw materials, kaolin, bauxite, waste materials, and/or fly ash as membrane precursors.

## 4. Cost Evaluation

Cost analyses of low-cost membranes are not presented in most of the published works on this topic. Nevertheless, it is worth mentioning that the fabrication of all the low-cost membranes presented here used lower sintering temperature relative to alumina. When one evaluates the economics of membrane fabrication, both raw materials and processing costs should be considered. A comprehensive cost breakdown is rarely presented in studies into membrane technologies [130]. According to some estimates, the cost of conventional ceramic membranes is in the region 500–1000 USD/m^2^ [190]. Costs of raw materials needed to fabricate a 1 m^2^ membrane from various starting materials are presented in Table 5. The table contains only literature with cost estimations to provide a comparison with conventional membrane materials. The cost advantages are significant relative to conventional ceramic materials of alumina, zirconia, and titania. Raw material costs ranging from 130 USD/m^2^ to 4 USD/m^2^ are estimated when naturally occurring clays are used as the main material for membrane fabrication [10,37]. Moreover, costs as low as 2 USD/m^2^ are possible when ash, a waste material, is used [122].

A meaningful economic analysis of membrane technology necessitates the consideration of externalities, namely the environmental impact of the extraction and processing of raw materials. Most of the membrane materials examined in this review incur a relatively minor environmental impact, and indeed the use of waste materials, such as fly ash or sugar cane bagasse, may constitute a positive externality by reducing the discharge of pollutants. Furthermore, the fabrication of membranes from ashes, clays, apatite, and quartz sand require relatively low sintering temperatures compared to alumina, zirconia, and titania. Cement and geopolymer require energy-intensive processing for the production of the raw materials used in membrane production, but the fabricated membranes themselves do not require sintering, and, overall, the fabrication of cement-based membranes is counted among the more environmentally friendly approaches to these systems.

## 5. Summary and Outlook

We have presented here a survey of diverse studies into the materials and approaches to the fabrication of low-cost water filtration membranes. The use of such low-cost membranes can enable the effective and large-scale treatment of industrial waste streams and oil-contaminated water. For each approach to membrane fabrication, numerous complexities need to be considered in the design of functional membrane systems. Parameters, including material composition, particle size distribution, the inclusion of additives, and pore formers, are just a few of the wide range of processing aspects that need to be taken into consideration in the production of effective low-cost filtration membranes. For such low-cost membrane technologies to play a significant role in alleviating global water shortages, several research pathways are identified here.

- The development of novel combinations of low-cost precursor materials and pore formers through iterative experimentation;
- Optimization of compositions and processing through machine learning-based methods [192,193];
- Continued development of freeze casting methods for the structuring of pores [194];
- Fabrication costs can further be reduced through the use of sintering-free materials [160,164];
- Safety concerns should be considered, i.e., effluents of low-cost membranes should be tested to check the presence and dissolution of radioactive elements and heavy metals [195].

The development of low-cost inorganic membranes from cheaply available precursor materials offers valuable prospects towards large-scale water treatment around the globe. There exist numerous emerging materials and methods that can be applied to achieve robust filtration membranes for water treatment without the disadvantages of polymeric membranes and the high costs typically associated with ceramic membrane technologies. For inorganic water filtration technologies to fulfill their full potential numerous aspects of design and fabrication, there is a need to be considered to achieve robust functional pore structures without incurring high processing costs or requiring significant energy input in sintering or densification. Of the various natural and waste materials surveyed in the present work, several points can be highlighted as a summary.

- Clays, in particular, are of keen interest in the design of low-cost ceramic membranes. Kaolin-based hollow fiber membranes, in particular, offer a valuable pathway towards effective oil-water separation;
- Silicate bearing ashes derived from coal combustion or rice husks can serve as the basis for mullite membranes in which the intergrowth of mullite needles is harnessed to impart highly functional pore structures in the obtained membranes;
- Current researches show that natural quartz sand, zeolite mineral, apatites are also promising. However, more research is needed to investigate the effect of fabrications conditions and mineralogical composition;
- A sintering-free approach using self-hardening materials, such as Portland cement and geopolymer, enables the reduction of costs and environmental impacts of high-temperature sintering processes;
- Cost-benefit analyses indicate that the application of low-cost materials in membrane processes on an industrial scale would be economically and environmentally advantageous.

## Figures and Tables

**Table 1:** Low-cost membranes prepared using kaolin as a main raw material.

Materials Mixed with Kaolin	Shaping Technique	Sintering Temperature, °C	Porosity, %	Pore Size, µm	Flexural Strength, MPa	Application
Quartz, sodium carbonate, calcium carbonate, and boric acid	Paste casting	850–1000	33–42	0.55–0.81	3–8	MF [20]
Quartz, calcium carbonate, sodium carbonate, boric acid, and sodium metasilicate	Pressing	900	35–39	0.72–1.69	7–11	MF of mosambi juice [30]
Quartz, calcium carbonate, sodium carbonate, boric acid, and sodium metasilicate	Pressing	900	30–37	2–3	-	MF of oil-in-water emulsions [31]
Quartz, ball clay, pyrophyllite, and feldspar	Extrusion	950	53	0.31	12	MF of oil in water emulsion [37]
Quartz and calcium carbonate	Pressing	900–1000	30	1.3	34	MF of oil and bacteria [11]
Limestone	Extrusion	800–1100	48	7	30	Support layer [38]
Lime	Extrusion	800–1100	47	8	30–53	Support layer [28]
Feldspar, sodium metasilicate nanohydrate, and boric acid	Pressing	850	29	0.93	8.7	MF [39]
Dolomite	Pressing and Extrusion	1000–1300	37–56	1.6–48	6–15	Support layer [40]
	Extrusion	1100–1300	44.6	4.7	47.6	Support layer [17]
Calcium carbonate	Extrusion	1250	52	4.0	23	Support layer [41]
	Extrusion	1150–1300	42–50	4–8	67–77	Support layer [42]
Calcite	Extrusion	1150	50	4	28	Support layer [19]
	Pressing and extrusion	1300 and 1100–1250	49	3	87	Support layer [18]
Bentonite, talc, sodium borate, and carbon black	Pressing	1000	34	0.65–1.25	58	MF of oil-in-water emulsion [32]
Bauxite	Pressing	1300–1600	31	0.15–0.8	100 *	MF [26]
Ball clay, quartz, alumina, and calcium carbonate	Paste casting	1100–1400	35–46	0.1–1	20–60	MF [29]
Ball clay, feldspar, calcium carbonate, and pyrophyllite	Pressing	800–1000	44	1.01	28	Support layer [12]
Alumina and aluminum hydroxide	Pressing	1300–1550	46	1.3	-	Support layer [27]
Without reactive additives	Support: extrusion; MF layer: slip casting	Support: 1000–1250; MF layer: 1050	46–60	0.9–1.4	4–24	MF [43]
	Extrusion	1150	49	1.2	5.8	Solid particle removal from water [36]
	Extrusion	1200–1500	-	0.32	221	Arsenic removal and oil removal [35,44]
	Extrusion	1100–1250	27	0.76	28	MF of cuttlefish effluent [34]
	Extrusion	1200–1500	32–57	0.53–4.25	15–35	MF of oil-in-water emulsion [25]
	Extrusion	1200–1500	-	0.4–0.5	70	MF of wastewater (oil and dye) [22]
	Pressing	1050–1100	43	0.5	20	MF [33]
	Support: pressing; UF: dip coating	Support: 900–1100; UF layer: 850–900	Support 30–41; UF 27	Support 1.4–6.3; UF 0.09	-	UF [23]
	Pressing	950	30	0.1	60	MF [21]

**Table 2:** Low-cost membranes prepared using natural clays.

Origin of Clay	Shaping Technique	Sintering Temperature, °C	Porosity, %	Pore Size, µm	Flexural Strength, MPa	Application
Argentina	Paste extrusion; slip casting	1000; 1200–1400	50	0.08–0.55	16–34	MF membrane [48]
Brazil	Pressing	1050	-	0.1–2	4–16	Water clarification from microalgae [49]
China	Dip coating	600	-	3–10 nm	-	Removal of phosphate ions [46]
	Pressing	1100–1350	-	1.4–1.9 and 10	45–69	Support layer [47]
	Paste casting	-	Above 50	10 nm	12.5	Oil-in-water emulsion filtration [50]
	Paste casting	-	Above 50	3.6–20 nm	28	Oily wastewater and protein separation [51]
	Paste casting	400	Above 60	12 nm	5–7	UF of oil-in-water emulsion [52]
India	Paste casting	800–1000	42	4.58	11.55	Removal of chromate [20]
	Paste extrusion	950	53	0.309	12	MF of oil-in-water emulsion [37]
Iran	Pressing	900	30	0.16–0.3	-	Removal of cationic dyes [53]
Morocco	Pressing	1000	25–40	0.01–1	-	Support layer [54]
	Paste extrusion	1250	43	11	10	Support layer [55]
	Paste extrusion	1250	-	-	-	Support layer [56]
	Pressing	700–1100	-	0.1–10	-	Support layer [57]
	Pressing	950–1250	-	0.3–1.8	-	Wastewater treatment [58]
	Extrusion	800	41	11	15	Support layer [59]
	Pressing	950	28–40	1.5–2.8	14	Support layer [60]
	Pressing	800–1050	32	1.2	22	Wastewater filtration [61]
	Pressing	850–1000	23–34	1.4–1.8	14.6	Support layer [62]
	Pressing	1100	28	2.5	17.5	MF membrane [63]
	Pressing	1000–1200	-	0.08, 0.6 and 3.8	-	Wastewater treatment [64]
NA	Pressing	1000–1100	36	0.29–0.67	27–32	MF of oil-in-water emulsion [65]
Nigeria	Pressing	1300	-	5–7 nm	7–18	UF of uranium from underground water [66]
Spain	Paste extrusion	850–1050	29–38	0.3–0.8	10–17	Support layer [67]
	Pressing or Paste Extrusion	1160	21–51	0.9–16	11–39	Support layer [68]
Tunisia	Paste and slip casting	1080; 900	49	SL: 6.3; MF: 0.18	-	MF of cuttlefish effluent [69]
	Slip casted	800	-	15 nm	-	UF of solution purification [70]
	Paste extrusion	900–1100	38	0.6–1.04	19	Support layer [71]

**Table 3:** Membranes fabricated using fly ash as a main raw material.

Fabrication Technique	The Particle Size of Fly Ash (Additives)	Sintering Temperature °C	Porosity, %	Pore Size, µm	Flexural Strength, MPa	Application
Extrusion	<10 µm	1100–1130	56–48	4.0–4.09	9.8–22.9	Support layer [117]
	-	1100–1500	30	0.5–1.0	8.5–85.8	Support layer [122]
Extrusion followed slip casting	15.41 µm; 5.01 µm; 1.41 µm	1190; 1150; 1000	-	2.13; 1.94 and 0.77	-	MF membranes [133]
Paste casting	-	900	42	0.885	43.6	MF of humic acid containing solution [126]
	-	800–1000	35–40	1.2	8–20	MF of oil-in-water emulsions [127]
Pressing	1.52 µm	1200–1550	35–45	0.93–2.2	22–65	Support layer [118]
	15.09 µm	1300–1500	39–44	6.52–7.28	28–36	Support layer [119]
	3.9 µm (bauxite 7.4 µm)	1200–1500	50	0.27–1.18	69.8	Support layer [120]
	11.94 µm (bauxite 5.66 µm)	1100–1500	52	0.67–1.78	34–87	MF of oil-in-water emulsion [121]
	2.1 µm (bauxite 1.2 µm)	1100–1500	48	0.18–0.26	81.2	Support layer [123]
	2.1 µm (bauxite 1.2 µm)	1100–1400	47.3	0.12–0.37	60–68	Support layer [124]
	1.14 µm, (dolomite 4.2 µm)	1100–1200	46	0.32	73	Support layer [125]
	2.53 µm (CaCO_3_ 9.15 µm)	1200–1350	49.6	0.5–1.2	34–90	Support layer [128]
	1–100 µm	1100	48	1.3–2.9	13	MF of oil-in-water emulsions [129]
	1–2.5 µm	1100	30–43	1.75–2.0	1.68–9.23	MF of oil-in-water emulsions [130]
	1–20 µm (mullite fiber)	800–1200	34	1–2	30	Support layer [134]
Slip casting	1 µm	800	51	0.25	-	MF of textile industry effluent [116]

**Table 4:** Comparison of oil separation ability of ceramic membranes prepared from low-cost and conventional materials.

Main Materials	Pore Size, µm	Oil Droplet Size, µm	Feed Concentration, mg/L	Removal of Oil, %
Clay	0.5	6.9	200	96 [179]
	0.65	2.84	100	96.7 [32]
	0.012	0.050–0.150	1350	97.4 [52]
Fly ash and bauxite	0.48	2	250	99 [121]
Fly ash and titania	0.11	1.1	200	97 [122]
Fly ash, quartz, and calcium carbonate	1.36	6.9	200	97 [130]
Fly ash, quartz, titania	1.32	6.9	200	99.2 [129]
Kaolin	1.42–0.35	12	-	90–100 [25]
Kaolin, ball clay	0.31	1.21	200	99.98 [37]
Kaolin, bentonite	<0.4	2.2	600	92.9 [65]
Kaolin, quartz	2.2	-	400	98.5 [31]
Kaolin, quartz, calcium carbonate	1.3	0.92	250	85 [11]
Sugarcane bagasse ash	1.8	-	-	99.9 [146]
Mullite-carbon nanotube composite	0.038	1.09	200	99.99 [180]
α-Alumina	0.05	-	500	97.7 [181]
Zirconia/α-alumina	0.2	1.79	1000	>97.8 [182]
Titania composite	0.9	-	200	99.56 [183]

**Table 5:** Raw material price for the fabrication of 1 m^2^ membrane, reported in the literature.

The Material Used for the Preparation of Membrane	Cost of Raw Material (USD)
Clay, sodium metasilicate, sodium carbonate, and boric acid	19 [20]
Fly ash quartz and calcium carbonate	5 [130]
Fly ash, calcium carbonate, sodium carbonate, and boric acid	17 [127]
Fly ash, quartz, calcium carbonate, and titania	25 [129]
Fly ash and titania	2 [122]
Kaolin, ball clay, feldspar, calcium carbonate, and pyrophyllite	10 [12]
Kaolin, quartz, ball clay, pyrophyllite, and feldspar	4 [37]
Kaolin, quartz, calcium carbonate	61 [11]
Kaolin, quartz, calcium carbonate, sodium carbonate, boric acid, sodium metasilicate, and polyvinyl alcohol	78 [30]
kaolin, quartz, calcium carbonate, sodium carbonate, boric acid, and sodium metasilicate	130 [10]
	135 [191]

## Abbreviations

AFt	Calcium sulfoaluminate hydrates
C2S	Dicalcium silicate
C3S	Tricalcium silicate
CH	Calcium hydroxide
C-S-H	Calcium silicate hydrate
MF	Microfiltration
PVA	Polyvinyl alcohol
RH	Rice husk
RHA	Rice husk ash
SCBA	Sugarcane bagasse ash
SEM	Scanning electron microscope
SL	Support layer
SSF	Slow sand filter
UF	Ultrafiltration
XRD	X-ray powder diffraction
